# Maternal Exposure to Carbamazepine at Environmentally Relevant Concentrations Causes Growth Delay in Mouse Embryos

**DOI:** 10.1021/acsomega.5c04235

**Published:** 2025-08-14

**Authors:** Eliane Veretnik, Orit Douek-Maba, Rotem Kalev-Altman, Aluma Haiman, Maxim Quint, Vered Mordehay, Neta Shlezinger, Yuval Cinnamon, Benny Chefetz, Dalit Sela-Donenfeld

**Affiliations:** † Koret School of Veterinary Medicine, The RH Smith Faculty of Agriculture, Food and Environmental Sciences, 108750 The Hebrew University of Jerusalem, Rehovot 76100, Israel; ‡ Department of Soil and Water Sciences, The RH Smith Faculty of Agriculture, Food and Environment, 72256 The Hebrew University of Jerusalem, Rehovot 7610001, Israel; § Department of Poultry and Aquaculture Science, Institute of Animal Sciences, 42718 Agricultural Research Organization - Volcani Institute, Rishon LeZion 7505101, Israel; ∥ Israeli Agriculture Research Organization- Volcani Institute, Rishon LeZion 7505101, Israel

## Abstract

The anticonvulsant drug carbamazepine is ubiquitous in the environment and has even even detected in human urine after consuming produce irrigated with reclaimed wastewater. Whether unintentional carbamazepine exposure through food and water affects public health is unknown. Its potential adverse effects are particularly concerning during pregnancy, as carbamazepine increases the risk of intrauterine growth restriction and congenital malformations in fetuses of carbamazepine-prescribed mothers. While environmental carbamazepine levels are much lower than clinical doses, its impact on early embryonic development, a period highly susceptible to malformations, requires investigation. This study used mice to examine the effect of exposing female mice to environmentally relevant carbamazepine concentrations (200/500/2000 ng/L in their drinking water) on embryos at gestation day 9.5. While no obvious malformations or compromised survival rates were observed, embryonic growth was delayed in a dose-dependent manner; developmental stages were younger than expected, fewer somites had formed, and heart maturation was delayed. Molecular analysis revealed a reduced expression of key developmental genes and decreased proliferation, linking growth delay to perturbed mechanisms. This study is the first to link maternal exposure to environmentally relevant carbamazepine concentrations with growth delay in mammalian embryos. Given that prenatal growth restriction contributes to human morbidity, this finding calls for further risk analyses of environmental pharmaceuticals on fetal health.

## Graphical abstract

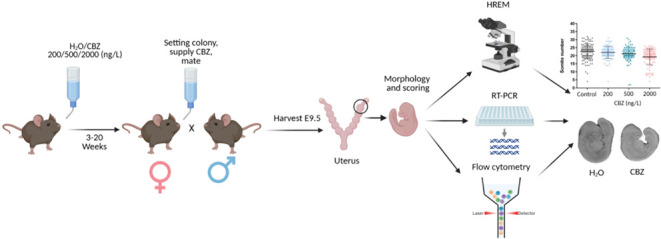

## Introduction

The perinatal period is highly vulnerable to adverse factors, particularly during the early embryonic stages when body systems are developing. Exposure to environmental contaminants during this critical developmental window can lead to a range of defects, including congenital malformations, neurological impairments, intrauterine growth restriction, and even pregnancy loss.^1–3^

Many psychoactive pharmaceuticals enter the environment via human excretion or industrial contamination.^4,5^ Among them, the anticonvulsant drug carbamazepine (Tegretol) is one of the most persistent pharmaceuticals found in effluents and treated wastewater.^6–12^ Moreover, following irrigation with treated wastewater, carbamazepine accumulates in soils and crops, enters the food chain, and reaches the population through dietary intake.^13–17^ Individuals residing in regions with the widespread use of treated wastewater are unknowingly exposed to carbamazepine from the environment.^18,19^ Although environmental concentrations of carbamazepine in treated wastewater are considerably lower than clinical levels in patients’ plasma (ng L^–1^ vs mg L^–1^),^11,14,20,21^ the potential impact of involuntary chronic exposure of the general population to environmentally persistent pharmaceuticals, like carbamazepine, warrants further investigation.

In perinatal medicine, carbamazepine is recognized as a teratogenic agent. Numerous epidemiological and clinical studies have shown that exposure to carbamazepine during pregnancy increases the risk of congenital malformations. The most frequently reported are neural tube defects (NTDs), followed—less commonly—by craniofacial anomalies, cardiac malformations, and urogenital abnormalities.^22–26^ Data from large pregnancy registries demonstrate a dose-dependent increase in the risk of major congenital malformations.^26^ In addition to structural abnormalities, several large population-based studies in the US and Europe have reported an association between carbamazepine exposure during pregnancy and increased rates of intrauterine growth restriction (fetuses small for gestational age) and microcephaly (head circumference below the population mean).^27,28^ These early growth impairments have been suggested to underlie disruptions in neurodevelopmental trajectories and cognitive deficits.^29–35^ Despite these findings, it is important to note that carbamazepine is considered to have a moderate teratogenic risk, especially when compared to other antiepileptic drugs such as valproate. Moreover, reported rates of congenital malformations vary across studies, likely due to differences in population characteristics and study design.^26,36^ Studies in rodents have also verified that embryonic development is disrupted by administering clinically comparable doses of carbamazepine to pregnant dams.^37–39^ Despite the known adverse effects of clinical doses of carbamazepine on mammalian embryos, it remains unclear whether exposure to carbamazepine from the environment poses a similar risk to human or other mammalian embryos.

Since experimental exposure of human embryos to carbamazepine is unethical, model systems are necessary. Several studies in fish have found that the addition of environmentally relevant concentrations of carbamazepine to the water tank led to a reduction in embryonic body length, delayed hatching, altered behavior, and a moderate increase in mortality,^40–48^ indicating the adverse effects of low carbamazepine concentrations in teleosts. Notably, because fish embryos lack an amniochorionic sac, they are directly exposed to carbamazepine from the water. This direct contact does not replicate the natural exposure route of mammalian embryos to pollutants, which occurs only via the mother. Therefore, the observed effects of environmental carbamazepine concentrations on fish may differ from those on amniote embryos, such as avian and mammalian embryos. By introducing carbamazepine at environmentally relevant concentrations into fertile chicken eggs, we have previously observed dose- and stage-dependent effects on the incidence of embryonic malformations, growth retardation, and mortality.^49^ While this finding demonstrated that avian embryos are susceptible to the adverse effects of environmental carbamazepine concentrations, direct introduction of carbamazepine into chicken embryos in the egg may also not accurately predict potential risks during in utero pregnancy in mammals.

This study utilized a rodent model system to determine whether administration of environmentally relevant concentrations of carbamazepine (200, 500, 2000 ng/L) in the drinking water of female mice affects their embryos’ development. Dose-dependent effects were observed on embryonic growth rate, cell proliferation, and gene expression. This study, which mimics a potential exposure route of human embryos to carbamazepine from the environment, is the first to demonstrate the susceptibility of mammalian embryos to involuntary, noninvasive exposure to environmental psychoactive drugs.

## Materials and Methods

### Carbamazepine Solution

Carbamazepine (99% purity, Sigma-Aldrich, Rehovot, Israel) was suspended in sterile tap water to prepare a stock solution of 20 ppm (20 mg/L), according to its reported maximum water solubility.^50^ The solution was sonicated for 15 min at 40 °C, stirred for 16 h at room temperature (RT) in a dark bottle, and stored at −20 °C before use. Further solutions were prepared with autoclaved drinking water obtained from the animal house facility to obtain working solutions of 200, 500, and 2000 ng/L. Water bottles were protected from direct light to avoid photodegradation of the pharmaceutical. To verify the precise concentration of CBZ in the water bottles, random samples from the water bottles were analyzed by a High-Resolution Mass Spectrometer (MS) Q-Exactive, with a Dionex RSLC system using a Kinetex 2.6 μm EVO C18 100A UHPLC column (Phenomenex, US). The MS was operated in positive ionization mode with an ESI Full Scan. Ion source parameters were as follows: spray voltage (+): 1300.00 V; capillary temperature: 256 °C; sheath gas: 51.00; aux gas: 3.00; sweep gas: 0.00; probe heater temperature: 413 °C. Data were analyzed using TraceFinder software. The limit of quantification was 0.25 ng/mL. Average carbamazepine concentrations of 207 ng/L in the bottles labeled as 200 ng/L, 500 ng/L in the bottles labeled as 500 ng/L, and 1873 ng/L in the bottles labeled as 2000 ng/L were measured (Figure S1). Notably, the rounded carbamazepine concentrations, rather than the exact measured values, were presented hereafter, as the rounded figures closely approximate the actual concentrations and did not affect the interpretation of the results while improving text readability,

### Mouse Procedures

C57BL/6J mice (Harlan Laboratories, Rehovot, Israel) were housed in a specific pathogen-free animal house with 12 h light/12 h dark cycles (7:00–19:00) under stable temperature (22 °C) and humidity (55%). At the onset of sexual maturity (6 weeks), females were randomly divided into different groups comprising 13–26 mice in each group. Two consecutive experiments were conducted in which females received standard drinking water or water containing environmentally relevant levels of carbamazepine. The first was a proof-of-concept experiment in which we tested a single concentration (500 ng/L) to determine whether any effect could be observed. Subsequently, we expanded the study into a dose–response experiment testing three different concentrations (200, 500, and 2000 ng/L). Both experiments were conducted using identical protocols, mouse strains, and experimental conditions. Different females from all groups in both experiments were exposed to carbamazepine ad libitum for 3–20 weeks before mating, as well as during gestation. The level of water in the water bottles was monitored to confirm uniform consumption across all experiments. As the average daily water consumption of a standard adult C57BL/6J female mouse is ∼4 mL,^51^ and as their average body weight typically ranges from 18–20 g, we estimated that the dams belonging to the 200/500/2000 ng/L carbamazepine groups ingested approximately 0.04 ng/g, 0.1 ng/g, or 0.4 ng/g body weight of carbamazepine each day, respectively.

For mating, each nulliparous female was housed with a single male for 16 h and examined for a vaginal plug the following morning, which was considered gestation day (GD) 0.5. On GD 9.5, females were euthanized, uteri were dissected, and embryos were collected into phosphate-buffered saline (PBS) (Biological Industries, Beit-Haemek, Israel) for further analyses. Altogether, all procedures were approved by the Hebrew University Animal Care Committee (ethical permit no: MD-20–16314–3, MD-23–17196–3).

### Analysis of Gestation Sacs, Embryonic Vitality, Stage, and Morphology

Each uterus was analyzed on gestation day (GD) 9.5 to confirm gestation and count the number of gestation sacs with viable/resorbed embryos. Embryonic sacs containing viable or resorbed embryos were distinguished by visual inspection: viable embryos were enclosed within rounded, translucent fluid-filled sacs that appeared as repetitive swellings along the uterine horns, whereas resorbed embryos were identified by shrunken, irregularly shaped, opaque, hemorrhagic sacs.

Embryos were dissected out of the sacs and visualized under a stereoscope (M165 FC fluorescent, Leica, USA) to relatively compare embryonic sizes within a litter, determine embryonic stage (E) at quarter-day intervals by well-defined anatomical milestones (Table S1), count somite pairs as a quantifiable, accurate, and continuous measure to determine sequential embryonic growth, and trace the sequential morphological transitions of the developing heart as an additional criterion for the embryonic growth rate (Table S2), all according to previous publications.^49,52–54^ All evaluations were conducted in a strictly blinded manner.

### Flow Cytometry

On GD 9.5, two embryos from the same litter were pooled to serve as a single biological replicate. A total of 5–7 biological replicates from 2 to 3 different litters per group were used for this analysis. Embryos were washed with PBS, dissociated into single cells by mechanical crushing through a 70m mesh membrane, and collected in FACS tubes. Cells were centrifuged at 400 *g* for 7 min and fixed in 4% paraformaldehyde (PFA) for 10 min at 4 °C. Following centrifugation and washing with PBS, cells were incubated in a permeabilization solution (PBS with 0.25% Triton and 1% bovine serum albumin (BSA)) for 10 min. Cells were recentrifuged, washed in PBS, and incubated in a blocking solution (PBS with 5% goat serum and 1% BSA) on ice for 30 min. Following another centrifugation and PBS wash, cells were incubated in a blocking solution containing an antihuman/mouse phospho-histone 3 (PhH3)-Alexa Fluor 488 conjugated antibody (1:300, Invitrogen, USA) for 30 min on ice. Cells were recentrifuged, washed with PBS, and analyzed using an Accuri C6 Flow Cytometer for fluorescence signal detection. As a blank control, cells underwent the same procedure without antibody staining. Data analysis was performed using the BD Accuri C6 software.

### High-Resolution Microscopy Analysis (HREM)

Embryos collected on GD 9.5 were fixed overnight (ON) in 4% PFA, washed with PBS, dehydrated in methanol, and impregnated with a 50% methanol:50% JB4 dye mix solution for another ON at 4 °C, followed by impregnation with 100% JB4 dye-mix solution for another ON at 4 °C. Prior to embedding, solution B (#14270–04, Electron Microscopy Sciences, Hatfield, PA, USA) was added to the JB4 dye mix for curing the resin, according to manufacturer’s instructions, as previously described.^49,54^ Sample blocks were mounted in the HREM machine (Serial no. 007, Indigo Scientific, Baldock, UK), serially sectioned at 1.74 μm, and imaged at 2700 × 1800-pixel resolution. The acquired HREM images were processed and stacked using Fiji software.^55^ 3D reconstruction was performed using Amira software (FEI, Oregon, USA), as previously described.^56^

### mRNA Purification, cDNA Preparation, and Real-time PCR

On GD 9.5, two embryos from the same litter were pooled to serve as a single biological replicate. A total of 3–4 biological replicates collected from 2 to 4 different litters were used for this assay. mRNA was collected using an mRNA extraction kit (Ambion, no. AM1914, USA) according to the manufacturer’s protocol, along with the RNase-Free DNase Kit (Norgen Biotek, no. #25710, Canada). RNA concentration and quality were measured using a NanoDrop 2000 spectrophotometer (Thermo Scientific, USA) and stored at −80 °C. Complementary DNA (cDNA) was prepared using a reverse transcription (RT) kit (Applied Materials, CA, USA), according to the manufacturer’s protocol, as previously described.^57^ RT was performed by incubating the samples for 10 min at 25 °C, 2 h at 37 °C, and 5 min at 85 °C, using a PCR device (Biometra, Germany), and cDNA was stored at −20 °C. Real-time PCR was performed using a StepOne Plus PCR system (Applied Biosystems, Norwalk, CA, USA), by mixing 3 μL of cDNA with 5 μL of SYBR Green PCR Master Mix Reagent (Thermo Fisher Scientific, USA), 2 μL of forward and reverse primers (10 μM), and 2 μL of DDW. The PCR program included 5 min at 95 °C and 40 cycles of 15 s at 95 °C and 45 s at 60 °C. Technical replicates were run in duplicates. As a negative control, cDNA was replaced with DDW. Results were normalized to the housekeeping gene Ubiquitin C (UBC) and analyzed using StepOne Software v2.2.2a (Applied Biosystems, USA) and a comparative ΔΔCT calculation. The following forward (F) and reverse (R) primers were used:

BRCA1: F’ AAGGAGCCCGTGTGCTTA, R’ TTGCCCTAGATGTGTTGTCTTTT;

BRCA2: F’ GCAGCACAGCAGATTTAGGAC, R’ CCGTGGGGCTTATACTCAGAT;

CDK2: F’ CCTTGCGGGTAAAGGCCAT, R’ CCTGCTTATCAATGCAGAGGG;

Cyclin D1: F’ GCGTACCCTGACACCAATCTC, R’ CTCCTCTTCGCACTTCTGCTC; HAND1: F’ CAAGCGGAAAAGGGAGTTGC, R’ GGTCTCACTGGTTTAGCTCCA;

Nanog: F’ AGGGTCTGCTACTGAGATGCT, R’ CAACACCTGGTTTTTCTGCCACCG; UBC: F’ CAGCCGTATATCTTCCCAGACT, R’ CTCAGAGGGATGCCAGTAATCTA;

OCT4: F’ CCTGGGCGTTCTCTTTGGAAAGGTG, R’ GCCTGCACCAGGGTCTCCGA; Sox2: F’ GCGGAGTGGAAACTTTTGTCC, R’ CGGGAAGCGTGTACTTATCCTT;

Sox9: F’ GCCACGGAACAGACTCACAT, R’ TGAGATTGCCcAGAGTGCTC.

### Data Analysis and Statistics

Data presentation and statistical analysis of the embryonic stage, number of somite pairs, and heart morphology scores were performed using R (version 4.4.1) within the RStudio IDE (version Cranberry Hibiscus) and the ggplot2 package (codes are displayed in Text S1). Statistical differences in embryonic stages, somite count, and heart scores were determined using one-way ANOVA for comparisons across multiple groups and Welch’s two-sample *t*-test for comparisons between two groups (i.e., control vs individual carbamazepine groups). Statistical pairwise correlations between heart score, embryonic stage, and somite number variables were determined by Spearman’s rank correlation coefficient analysis via R (Table S3). Analysis and presentation of the real-time PCR and flow cytometry data were performed using GraphPad Prism 8 software. Statistical differences in mRNA levels and fluorescence intensity were determined using one-way ANOVA and two-sample *t*-test. Differences were considered significant at *p* < 0.05.

## Results and Discussion

### Exposure of Female Mice to Environmentally Relevant Carbamazepine Concentrations Does Not Impair Their Ability to Gestate

To investigate whether environmentally relevant concentrations of carbamazepine could potentially compromise the development of mammalian embryos, we utilized mouse embryos as a common preclinical model organism, closely mimicking the key aspects of human embryogenesis.^58^ To accurately replicate a long-term and noninvasive exposure route of human embryos to environmental contaminants via maternal transfer, female mice were provided with standard drinking water or drinking water supplemented with 200 ng/L, 500 ng/L, or 2000 ng/L carbamazepine, concentrations representing levels frequently encountered in the environment.^7,12,16^ Female mice were exposed for varying durations (3–20 weeks) to evaluate whether the duration of exposure could exert specific effects on embryonic development. A minimum exposure period of 21 days was strategically chosen based on previous research that detected isotope-labeled carbamazepine within mouse embryos following exposure of the dams to a concentration of 100 μg/L for 20 days.^59^ Following mating, uteri were extracted on gestation day (GD) 9.5. As a primary step, we determined whether exposure to environmentally relevant levels of carbamazepine impacts the ability of female mice to gestate or facilitate embryonic implantation. Uteri from control females (*n* = 26) and carbamazepine-exposed females (*n* = 14 for 200 ng/L, *n* = 16 for 500 ng/L, *n* = 13 for 2000 ng/L) were analyzed on GD 9.5 (Figure 1). All experimental groups exhibited an average of 8–9 gestation sacs, with no statistically significant differences between groups (*p* > 0.5, one-way ANOVA). The majority of the gestation sacs contained viable embryos, while ∼1–2 sacs occasionally contained resorbed embryos (Figure 1A–E). These results demonstrate that exposing dams to a range of environmentally relevant concentrations of carbamazepine does not compromise their ability to sustain pregnancy, enable embryonic implantation, or alter the overall litter size.

**Figure 1:**
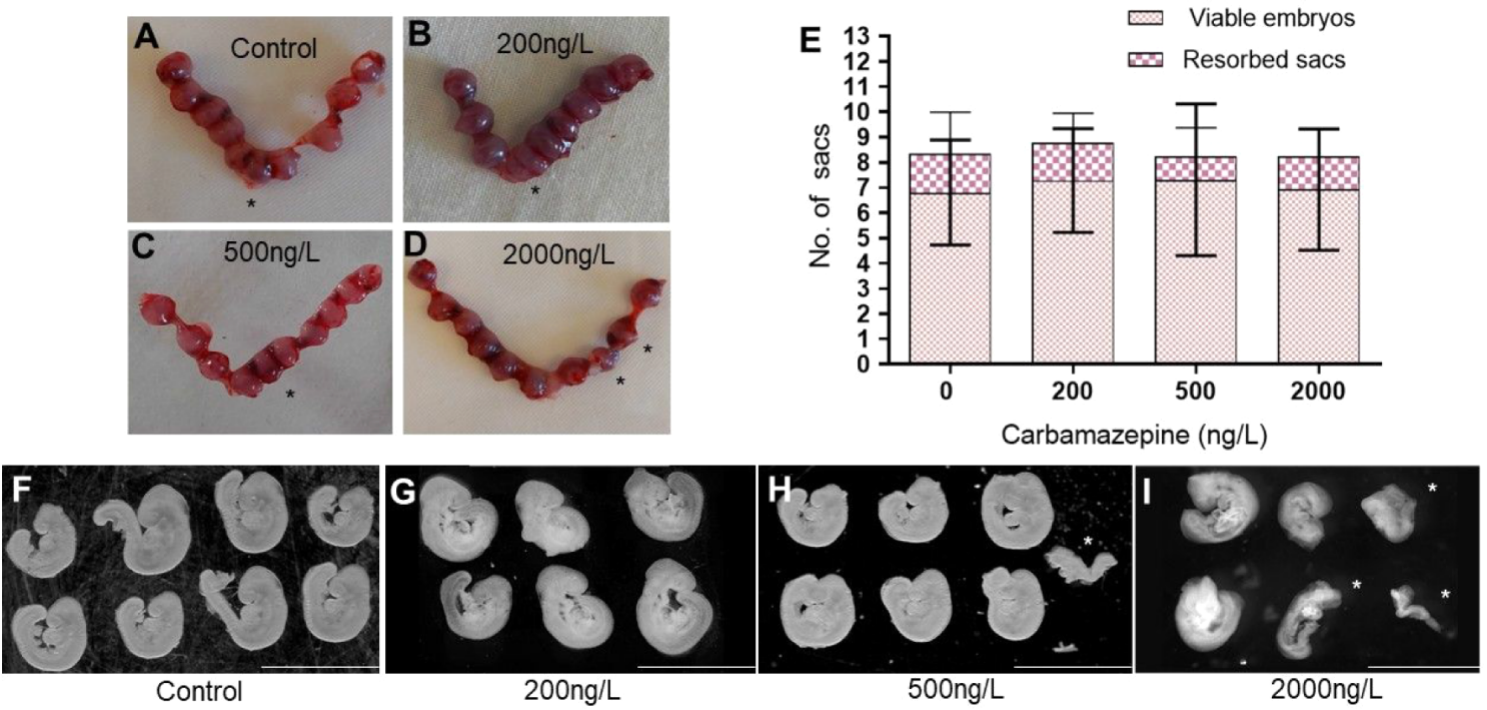
Effect of environmentally relevant concentrations of carbamazepine on gestation, litter size, embryonic mortality, and gross morphology. (A–D) Representative images of uteri from control and carbamazepine-exposed females on GD 9.5, showing gestation sacs. Resorbed sacs are marked with asterisks. (E) Quantification of gestation sacs with viable or resorbed embryos per uterus across groups: control (*n* = 26), 200 ng/L (*n* = 14), 500 ng/L (*n* = 16), and 2000 ng/L (*n* = 13) carbamazepine concentrations. No statistically significant differences were found between groups (viable embryos, *p* = 0.8869; resorbed sacs, *p* = 0.5422, one way ANOVA). (F–I) Representative images of embryos from control and carbamazepine-exposed groups collected from a single uterus on GD 9.5. Smaller-than-expected embryos are marked with asterisks. Carbamazepine concentrations are indicated. Scale bar in (F–I) = 3.7 mm.

### Environmentally Relevant Carbamazepine Concentrations Induce Embryonic Growth Delay Dose-Dependently

Following uterine analysis, we proceeded to harvest the embryos and examine their gross morphology. In total, 147/95/81/102 embryos from 26/14/16/13 females in the control, 200, 500, or 2000 ng/L carbamazepine groups, respectively, were analyzed. Most embryos within each litter displayed the expected embryonic (E) stage of 9.5 (E9.5), as recognized by the standard Theiler embryonic staging criteria for this time point,^52^ with no apparent malformations.^49,56^ However, while all embryos from the same litter were imaged under identical magnification conditions, relative size differences were observed between embryos, with some appearing slightly or substantially smaller than their littermates (Figure 1F–I, asterisks). Random deviations from the expected stage are known to occur within litters, potentially resulting from variations in conception time, implantation site, and nutrient availability.^60–62^ Nevertheless, prior studies have also linked stage variations to maternal exposure to pollutants.^63–66^ Therefore, we aimed to systematically determine whether the observed growth differences were random or associated with different carbamazepine concentrations.

Embryonic growth was first determined by combining the well-established Theiler atlas for mouse embryonic staging, which provides standardized morphological landmarks at quarter-day intervals (Table S1), together with a recent single-cell atlas that presents high-resolution morphological changes at 2–6 h intervals.^52,53,67^ In total, 147/95/81/102 embryos from 26/14/16/13 females in the control, 200 ng/L, 500 ng/L, or 2000 ng/L carbamazepine groups, respectively, were analyzed. The mean values of the embryonic stages in the control and the 200 ng/L groups were E9.48 and E9.42, respectively (*t*-test, *p* = 0.2888 for 200 ng/L vs. control, ns). Embryos from the 500 ng/L and 2000 ng/L groups demonstrated a slightly younger stage, with mean values of E9.29 and E9.22, respectively (*t*-test, *p* = 0.0015 for 500 ng/L vs. control, and *p* < 0.0001 for 2000 ng/L vs control). These results indicate a significant dose-dependent decrease in the embryonic stage upon maternal exposure to carbamazepine (Figure 2A, one-way ANOVA: *p* < 0.0001).

**Figure 2:**
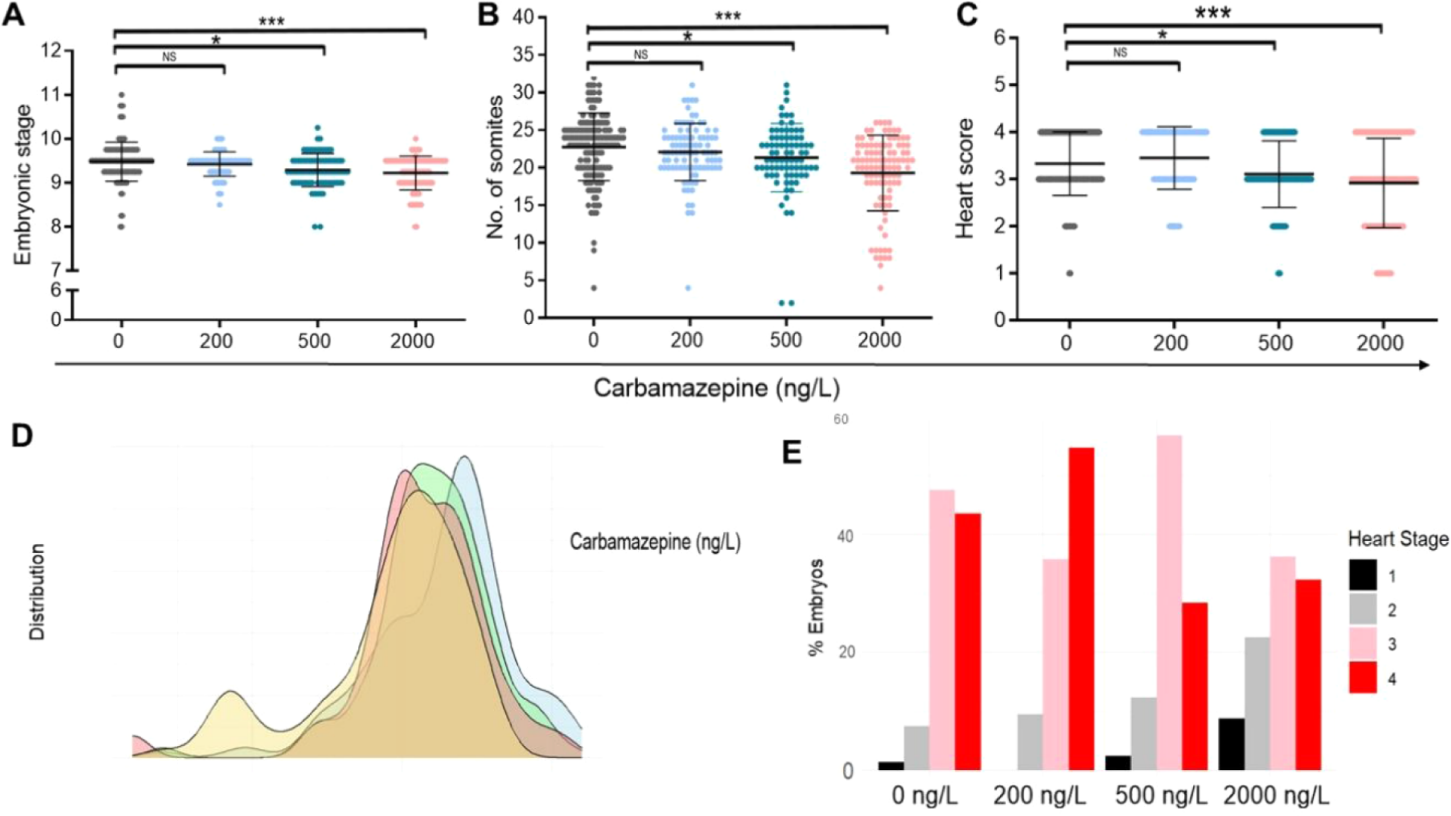
Environmentally relevant carbamazepine concentrations induce embryonic growth dose-dependently. Analysis of embryonic stage (A), number of somites (B), and heart morphology (C) in individual embryos from control and carbamazepine-exposed groups on GD 9.5. Each dot represents an embryo, and scores in (C) indicate heart developmental stages. (D) Smoothed-density plot showing the spatial distribution of somite numbers across control and carbamazepine-exposed embryos. The color bar indicates each group. (E) Distribution of heart scores across all groups, with the color bar marking each score. Control: 147 embryos/26 dams; carbamazepine 200 ng/L = 95 embryos/14 dams; 500 ng/L = 81 embryos/16 dams; 2000 ng/L = 102 embryos/13 dams. Statistical significance: *p < 0.05, **p < 0.01, ***p < 0.001. Supporting Information provides R-codes and heart score details.

To further measure embryonic growth in a more quantifiable manner, the somite number was counted (Figure 2B). Somites, the precursor structures of the vertebrae and axial muscles, serve as accurate, objective, and reproducible criteria to determine embryonic progress, as somite pairs form sequentially at regular 2 h intervals and reflect the embryo’s precise morphological stage.^58,62,68^ Somite count revealed an average of 22.77 and 22.08 somites in the control and 200 ng/L groups, respectively (*t*-test, *p* = 0.2217, ns). However, the 500 ng/L and 2000 ng/L groups displayed fewer somites, averaging 21.33 and 19.31 somites, respectively (*t*-test, *p* = 0.0226 for 500 ng/L vs control, and *p* < 0.0001 for 2000 ng/L vs control), indicating a significant dose-dependent decrease in somite count upon exposure to carbamazepine (Figure 2B, one-way ANOVA: *p* < 0.0001). Data analysis by a smoothed density plot further illustrated the gradual shift toward lower somite counts with maternal exposure to increasing levels of carbamazepine (Figure 2D and Text S1). Notably, the reduced developmental stage (Figure 2A) and somite number (Figure 2B) did not correlate with the female’s exposure time to carbamazepine (Figure S2); control females or females exposed to different carbamazepine levels for varying durations mostly yielded embryos with similar stage and somite variations as embryos from the other groups, with no significant increase in growth delay with longer exposure time (except for some variants, which are expected due to natural differences). Collectively, these results indicate a dose-dependent delay in early embryonic growth following maternal exposure to environmentally relevant carbamazepine concentrations. Although the delayed growth is relatively mild, this effect is consistent and statistically significant.

To further validate the impact of carbamazepine on embryonic growth, heart development was also evaluated. The heart, which is the first functional organ in the embryo, undergoes a tightly ordered sequence of morphogenetic events from E8 to E10. While at E8 a primary linear heart tube appears in the midline and begins to pump blood, as development proceeds, the heart begins looping rightwards at E8.5, followed by gradual elongation and regionalization at E9.0–9.5, until forming a complex four-chambered structure with specialized subregions and cell layers at E10 (Table S2).^69^ As such, any delay in heart development can be visually noticed. Given that carbamazepine has been previously shown to impair cardiac cell function in vitro,^70^ we assessed heart morphology based on its well-established developmental landmarks (Table S2).^71,72^ Hearts were scored from 1 to 4 according to their morphological state at the time of analysis (Figure 2C and Table S2). Mean heart scores for embryos from the control and 200 ng/L carbamazepine groups were 3.329 and 3.421, respectively (*t*-test, *p* = 0.1784, ns), whereas the 500 ng/L and 2000 ng/L groups displayed lower heart scores, averaging 3.111 and 3.032 (*t*-test, *p* = 0.0203 for 500 ng/L vs control, and *p* < 0.0001 for 2000 ng/L vs control). These results indicated a significant dose-dependent delay in heart development (Figure 2C, one-way ANOVA, *p* = 0.0008). Global analysis of heart score’s distribution further demonstrated the gradual shift from higher to lower heart scores in embryos exposed to 500 and 2000 ng/L carbamazepine compared to the control and 200 ng/L exposed groups (Figure 2E and Text S1). Overall, these analyses suggest that passive exposure of mouse embryos to increasing doses of carbamazepine at environmentally relevant levels results in developmental delay, characterized by a higher proportion of embryos with fewer somites, earlier-than-expected embryonic stage, and delayed heart maturation.

We next investigated whether the observed growth retardation was coordinated across all parameters, as analyzed within individual embryos. This is an important distinction because the health implications of a generally smaller, but otherwise normal, embryo differ significantly from those of an embryo with a developmental defect, where the growth of different organ systems is disproportionate.^73,74^ To distinguish between these possibilities, we integrated data from the somite count, embryonic stage, and heart scores into a multidimensional plot (Figure 3 and Text S1). The analysis revealed a consistent correlation across all experimental groups: the number of somites (represented on the *Y*-axis) directly corresponds with both the heart developmental score (shown on the *X*-axis) and the embryonic stage (indicated by color intensity), demonstrating a synchronized progression of these developmental parameters, which was proportionally delayed with increased carbamazepine concentrations. Specifically, embryos with higher somite counts tended to cluster with those exhibiting higher heart scores and a more advanced embryonic stage, while embryos with fewer somites clustered with those displaying lower heart scores and a less advanced stage (i.e., Figure 3A,B vs C,D, bottom left). Spearman’s correlation analysis confirmed statistically significant positive correlations between the measured parameters across all experimental groups (Table S3, *p* < 0.0001). These data indicate that the embryonic stage, somite count, and heart developmental scores progress in a coordinated manner—not only in the control group but also in all CBZ-exposed groups, even in cases of delayed embryonic growth, reinforcing the conclusion of our multiparametric analysis (Figure 3). Notably, the strongest correlations were found between somite count and stage, while the most moderate correlations were observed between somite count and heart score (Table S3), suggesting that the somite number and embryonic stage closely reflect developmental progression, whereas heart development may advance with slightly more variability. Altogether, given that the reduction in overall embryonic growth is well-coordinated upon exposure to carbamazepine, it is suggested that the observed developmental delay induced by carbamazepine is not associated with pronounced embryonic malformations. Moreover, this analysis indicates that the most developmentally advanced embryos were exclusively found in the control group (Figure 3A, top right), raising the possibility that even a lower concentration of carbamazepine than 500 ng/L could induce a subtle growth delay.

**Figure 3:**
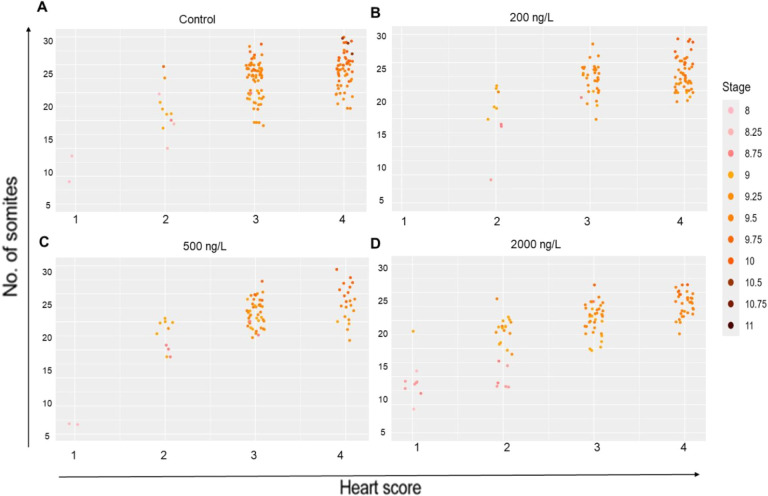
Multidimensional scatter plot to integrate the data from all analyzed parameters in the individual embryos. Visualization of embryonic distribution by integrating the data from the somite count (*Y*-axis), heart score (*X*-axis), and embryonic stage (color intensity) in control (A) and carbamazepine-exposed embryos (B–D). Each dot represents one or more clustered embryos. Heart score and somite counts are indicated on the *X* and *Y* axes, respectively. The color intensity, indicated by the bar on the left, correlates with embryonic stage progression. Supporting Information provides R-codes and heart score details.

Even though the growth-delayed embryos showed no obvious malformations, identifying subtle congenital defects at E9.5 based solely on gross morphology can be challenging.^75^ Therefore, HREM scanning was used to analyze representative embryos from the control and 2000 ng/L carbamazepine groups (Figure 4). This 3D imaging platform provides unprecedented detail of external and internal embryonic structures, surpassing that of standard light microscopy modalities.^57,76,77^ HREM analysis using 3D lateral views and digital frontal sections (Figure 4A,B or C–G, respectively) revealed a clear developmental delay in the carbamazepine-exposed embryo compared to the control. For example, while the control embryo reached 22 somites, the carbamazepine-exposed embryo developed to only 18 (Figure 4A,B, asterisks). Despite this difference, the somites exhibited no abnormalities in structure, size, or symmetrical distribution along the body axis. Forelimb bud and pharyngeal arche morphology also differed, with the control embryo showing larger and more advanced structures compared to the carbamazepine-exposed embryo (Figure 4A,B). However, the overall shape and position of these tissues appeared normal in the growth-delayed embryo. Frontal sections through the trunk region (somites 7–11) revealed further disparities (Figure 4C,D). In the control embryo, sclerotomes (cartilage and bone precursor cells) were more condensed, and primary myotomes (progenitors of the axial muscles) were clearly formed (Figure 4C, red asterisks and arrowheads, respectively). In contrast, the carbamazepine-exposed embryo exhibited less condensed sclerotomes and minimal myotome formation (Figure 4D, red asterisks), indicating delayed somite differentiation. Posterior views of the tail region also revealed differences in neural tube development (Figure 4E,F). The caudal neuropore in the control embryo was nearly closed (Figure 4E, black arrow), whereas it remained wide open with an extended neural groove in the carbamazepine-exposed embryo (Figure 4F, black and yellow arrows), features characteristic of less-developed neural tubes. Finally, examination of the heart frontal sections revealed clear differences in cardiac development. In the control, the myocardial and endocardial layers were well-defined, and trabeculae carneae were evident in both atria and ventricles (Figure 4G, red arrows). Conversely, the carbamazepine-exposed embryo displayed a thinner myocardial layer, smoother inner ventricular and atrial surfaces, and absent trabeculae carneae (Figure 4, arrows). Nevertheless, subdivision of the heart and initial outflow tract formation appeared normal. These HREM analyses reinforce our findings that exposure of female mice to 2000 ng/L carbamazepine induces a notable growth delay in the embryos without causing malformations on GD 9.5. This suggests that, apart from embryonic growth retardation (EGR), carbamazepine at these concentrations does not induce severe defects in mammalian embryos, unlike its teratogenic effect at clinical doses.^23,30,78^ However, the possibility of later-onset congenital defects cannot be excluded.

**Figure 4:**
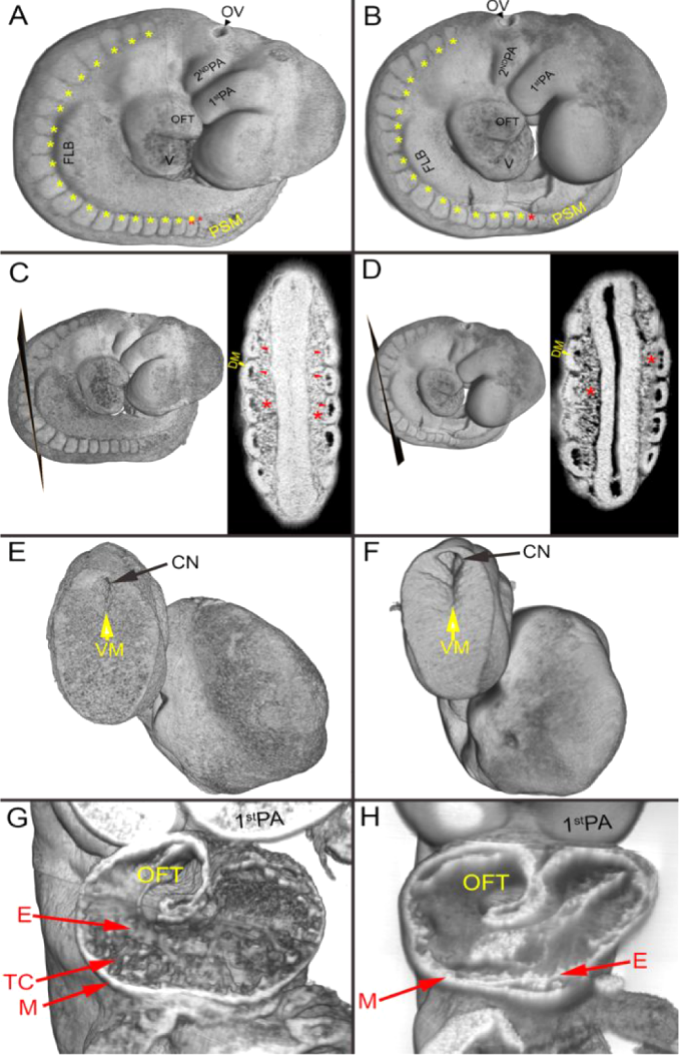
3D HREM modeling reveals developmental differences between control and carbamazepine-exposed embryos. Side views of whole embryos from control (A) and carbamazepine (B) groups. Yellow asterisks denotes all somites except the last, which is marked in red. Labeled structures include forelimb bud, first/second pharyngeal arches, otic vesicle, heart outflow tract, heart ventricle, and presomitic mesoderm. Frontal digital sections through somites 7–11 in control (C) and carbamazepine-exposed (D) embryos. Dermomyotomes are marked, and sclerotomes are labeled with red asterisks. Posterior views of the tail region in control (E) and carbamazepine-exposed (F) embryos, highlighting the caudal neuropore and ventral midline. Frontal digital sections through the heart of control (G) and carbamazepine-exposed (H) groups. Indicated structures include myocardial and endocardial layers, trabeculae carneae, outflow tract, and first pharyngeal arch. Abbreviations: first PA: first pharyngeal arch, 2nd PA: second pharyngeal arch, CN: caudal neuropore, DM: dermomyotome, E: endocardium, FLB: forelimb bud, M: myocardium, OFT: outflow tract, OV: otic vesicle, PSM: presomitic mesoderm, V: ventricle, VM: ventral midline of the neural tube, TC: trabeculae carneae.

Our findings align with zebrafish studies showing delayed embryonic hatching following exposure to low doses of carbamazepine, an effect associated with dose-dependent impacts on reproduction, behavior, morbidity, and mortality.^41,42,44,79–81^ Similarly, our prior work with chick embryos demonstrated a negative, dose-dependent effect of environmentally relevant levels of carbamazepine on their growth.^49^ However, the growth delay in chick embryos was more pronounced than that in mice and was sometimes accompanied by malformations and mortality. These species-specific differences may be due to the contrasting exposure routes. Unlike mouse embryos, which are exposed indirectly, zebrafish and chick embryos experience direct exposure to carbamazepine via the surrounding water or egg,^40–49^ potentially resulting in higher concentrations of the drug contacting their developing body systems. Furthermore, given that previous studies in fish, mice, and cultured cells have reported more significant effects with higher environmentally relevant doses of carbamazepine than those used here,^59,70,81–85^ it is possible that more severe effects will be detected in mouse embryos with maternal exposure to higher carbamazepine concentrations than those used here.

The varied effects of carbamazepine at environmentally relevant levels on different species may also be influenced by its metabolism; carbamazepine metabolism in rodents is much faster than in zebrafish (half-life: ∼1–4 h versus ∼12 h, respectively) and in humans (half-life: ∼12–24 h during chronic use).^86–89^ The species-specific carbamazepine metabolism may describe the milder developmental defects observed in mouse embryos compared with those reported in zebrafish, as rapid metabolism limits accumulation of carbamazepine and facilitates its detoxification. Likewise, although carbamazepine pharmacokinetics have not yet been directly studied in avian species, the catalytic activity and expression levels of CYP3A enzymes have been reported to be lower in birds than in rodents.^90^ These differences may similarly contribute to the milder developmental outcomes observed in mouse embryos compared to chick embryos that were exposed to similar carbamazepine concentrations.^49^ Given that interspecies variations in carbamazepine pharmacokinetics are critical for assessing its teratogenic effects across model organisms, further research is essential to determine the precise concentrations of carbamazepine and its metabolites in various embryonic species, as well as their comparable teratogenicity.

In accordance with our observation of mild growth restriction in mouse embryos exposed to environmentally relevant concentrations of carbamazepine, a more significant reduction in fetal weight and length was reported in rodents exposed to clinically relevant doses of carbamazepine.^37–39,59,70,81–85^ These effects were accompanied by other malformations and endocrine disruptions, supporting a dose-dependent disruption of carbamazepine on mouse embryonic growth. Importantly, numerous meta-analyses in humans have confirmed that embryonic growth restriction (EGR) is a potential outcome in pregnancies of women treated with carbamazepine. Carbamazepine was found to be associated with a modest reduction in birth weight and an increased risk of small for gestational age (SGA) in a dose-dependent manner.^26,91^ Likewise, a large prospective NEAD (Neurodevelopmental Effects of Antiepileptic Drugs) study showed that in utero exposure to carbamazepine was associated with an increased rate of SGA and microcephaly at birth, although head size normalized by 24 months.^27,92^ Yet, the transient smaller-than-average head size was suggested to reflect neurodevelopmental abnormalities that may induce higher rates of behavioral and learning disorders and cognitive deficits in these children.^31,35^ Nevertheless, the more recent MONEAD (Maternal Outcomes and Neurodevelopmental Effects of Antiepileptic Drugs) study did not find such a correlation.^36,93^ The differences between the NEAD and MONEAD study findings on fetal growth are likely to stem from changes in clinical practice of carbamazepine administration over time, which may reduce adverse outcomes, study design differences, population differences, and differences in how growth outcomes were defined and measured. Nevertheless, these apparent discrepancies underscore the importance of drug-specific risk evaluation when managing epilepsy in pregnancy, the understanding of confounding factors (i.e., maternal health, nutrition, seizure control, smoking) that may affect fetal outcomes, and the unraveling of whether a moderate increase in FGR is also evident in regions where carbamazepine is commonly detected in the environment.

### Environmentally Relevant Carbamazepine Levels Alter Gene Expression

Embryonic growth relies heavily on controlled cell division, a process in which the cell cycle progresses through distinct phases: G1 (gap 1), S (DNA synthesis), G2 (gap 2), and M (mitosis). A complex network of cell cycle genes coordinates these phases to ensure proper tissue growth and differentiation. Dysregulation of these genes is frequently associated with delayed embryonic growth, mortality, and/or congenital defects.^94–96^

One potential mechanism by which carbamazepine induces EGR is through its effect on cell cycle genes. To investigate this, mRNA was extracted from embryos of all experimental groups to assess the expression of selected genes (Figure 5). We examined two master regulators of the cell cycle: cyclin D1 and cyclin-dependent kinase 2 (CDK2). These genes facilitate the G1/S transition into the S phase and promote DNA replication.^97–99^ A significant reduction in their expression levels was found in embryos from the 500 and 2000 ng/L carbamazepine groups compared to the control and 200 ng/L carbamazepine groups (Figure 5A,B). These findings are consistent with knockout studies in mice showing that cyclin D1- or CDK2-deficient embryos/neonates are smaller than their wild-type littermates. In addition, cells extracted from these mice exhibited delayed proliferation.^100,101^ We also investigated the expression of two additional cell cycle regulators, the breast cancer susceptibility genes BRCA1/2, which govern DNA repair during cell division.^102–104^ In contrast to cyclin D1/CDK2, their expression did not differ significantly between the groups (Figure 5C,D). This result suggests that the effect of carbamazepine on the cell cycle is not primarily induced by DNA damage, as this would typically result in upregulation of these genes.^105^ Notably, the fact that carbamazepine did not alter the expression of all examined genes argues against a nonspecific/toxic effect on global gene transcription.

**Figure 5:**
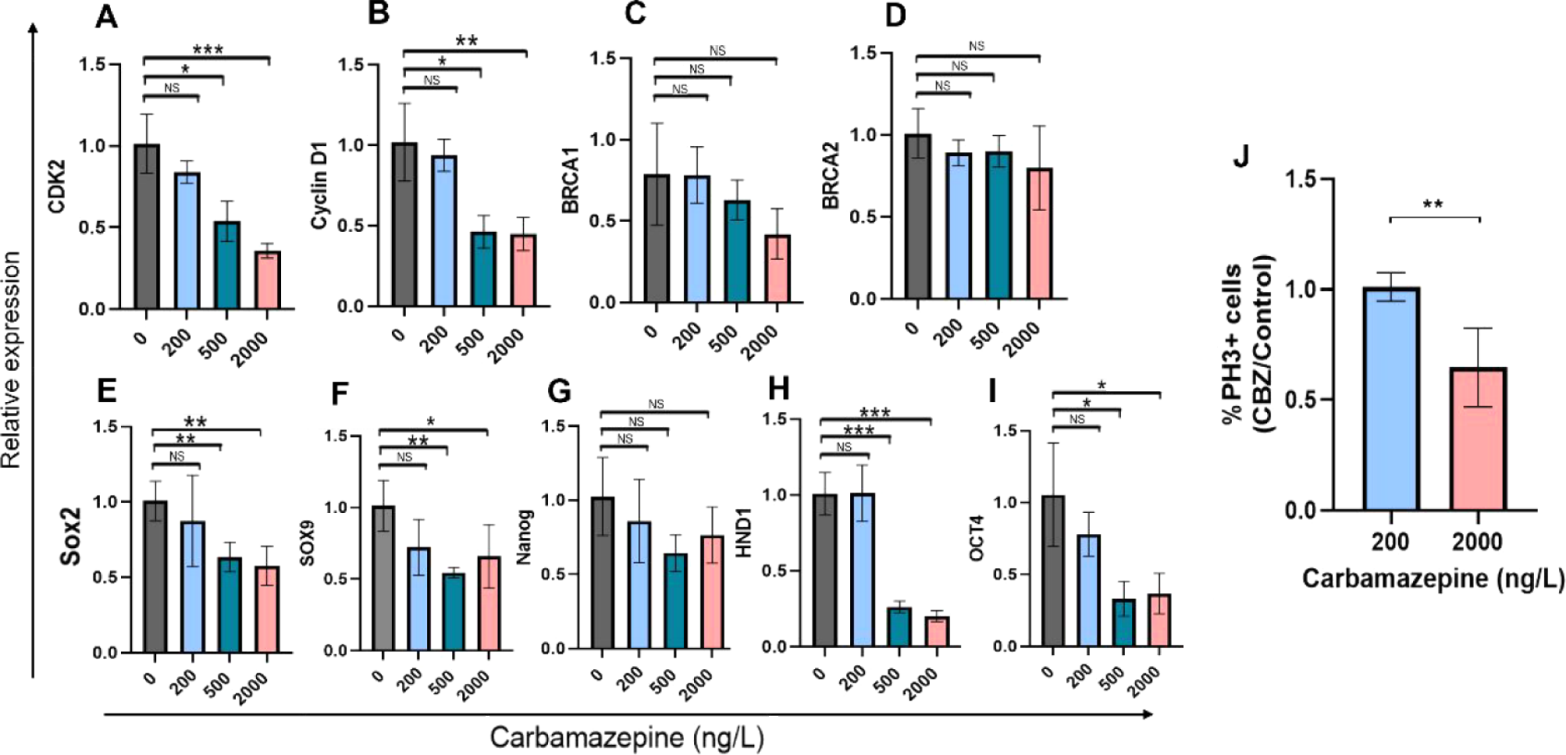
Maternal exposure to environmentally relevant carbamazepine levels alters embryonic gene expression. (A–I) Gene expression analysis by real-time PCR on mRNA extracted from embryos in all groups. Each bar represents data collected from 6 to 8 embryos from 3 to 4 dams. (J) Flow cytometry quantification of PhH3-expressing cells in carbamazepine-exposed embryos in relation to controls. Each bar represents data from 5 to 7 embryos from 2 to 3 dams. Statistical significance: *p < 0.05, *p < 0.01, ***p < 0.001 (one-way ANOVA).

To further examine the effect of carbamazepine on cell division, embryos from the control, 200 ng/L, and 2000 ng/L carbamazepine groups were collected on GD 9.5, separated into single cells, and immuno-stained for phospho-histone H3 (PhH3), a specific marker for cells in the M phase of the cell cycle.^54,106,107^ Flow cytometry was used to quantify the percentage of PhH3-positive cells in the control vs carbamazepine-exposed samples. The percentage of PhH3-expressing cells was significantly lower in the 2000 ng/L carbamazepine group compared with the 200 ng/L group (Figures 5J and S3). This finding indicates that the developmental delay induced by maternal exposure to 2000 ng/L carbamazepine is associated with a reduction in the number of actively dividing cells, consistent with the observed decrease in cell cycle gene expression. This result also aligns with our previous findings of decreased cell division in chick embryos exposed to environmentally relevant concentrations of carbamazepine.^49^ Likewise, studies using mammalian cell lines have reported that carbamazepine treatment can lead to cell cycle arrest, altered cell-cycle gene expression, and impaired centrosome separation, although these studies employed higher carbamazepine concentrations.^108–111^ Despite this evidence, the precise molecular mechanism(s) by which carbamazepine affects cell division remains unclear.

We also assessed the expression of key regulators of early embryonic development. First, we examined *nanog* and Oct4, two core genes essential for cell pluripotency and self-renewal in embryonic stem cells.^112–114^ *Oct4* mRNA levels were significantly downregulated in embryos from the 500 and 2000 ng/L carbamazepine groups (Figure 5I). In contrast, *nanog* expression did not significantly differ between the control and any of the carbamazepine groups, although a trend toward decreased expression was observed in the 2000 ng/L group (Figure 5G). These results further indicate that the effect of carbamazepine on gene expression is target-specific rather than global. This target specificity is consistent with the lack of severe embryonic malformations, which would be predicted if both *Oct4* and *nanog* were downregulated. Interestingly, Oct4 was found to regulate cell-cycle progression via modulating *cyclin/CDK* expression,^114^ indicating a plausible link between the downregulation of *Oct4* and that of *cyclin D1/CDK2* in the affected embryos.

Next, we analyzed the expression of members of the Sox family of transcription factors, Sox2 and Sox9. These genes play critical roles in neuronal and mesodermal lineage specification, respectively, by regulating multipotency, proliferation, and/or differentiation in stem cells and neural and mesenchymal tissues.^115–117^ The expression of both Sox2 and Sox9 was downregulated in the 500 and 2000 ng/L carbamazepine groups compared to the control and 200 ng/L carbamazepine groups (Figure 5E,F). These results suggest that the EGR may be associated with the downregulation of *Sox2/9,* leading to delayed development of neural and mesodermal cell lineages, as evidenced by the lower somite number, less developed limb bud and pharyngeal arches, and delayed somite and neural tube maturation (Figures 3–5).

Finally, as carbamazepine also delayed heart development (Figures 3–5), we investigated the expression of *Hand1,* an essential factor in early cardiac development.^118,119^ *Hand1* mRNA levels were reduced in the 500 and 2000 ng/L carbamazepine groups compared to the control and 200 ng/L groups (Figure 5H), suggesting a potential link between lower *Hand1* levels and slower heart development. Noticeably, while carbamazepine-exposed embryos exhibited younger-than-expected hearts without obvious heart malformations (Figure 4H), Hand1-null mice displayed embryonic lethality at E8.5 due to cardiac defects.^120^ The milder effect of carbamazepine on cardiac development in our system, compared to Hand1 mutant mice, raises the possibility that Hand1 downregulation is compensated by other genes not altered by carbamazepine, as demonstrated in other studies.^61,121–123^ Altogether, these results suggest that maternal exposure to carbamazepine affects genes involved in various developmental processes in addition to its effect on cell division. As no severe malformations were observed in the growth-delayed embryos, it is possible that the reduction in gene expression levels observed here is reversible and/or rescued by other, nonimpaired, genes. However, we cannot exclude the possibility that these alterations in gene expression could trigger malformations that are not yet apparent at E9.5. Finally, emerging evidence suggests that carbamazepine can participate in global epigenetic regulation by modifying chromatin structure and transcriptional activity.^124–126^ Although this activity has so far been reported only under clinical and subclinical concentrations, further research is needed to elucidate whether environmentally relevant concentrations of carbamazepine may also modulate gene expression via epigenetic regulation.

### Possible Implications on Human Health

Our data consistently demonstrate a dose-dependent, mild growth delay in embryos from mothers exposed to environmentally relevant concentrations of carbamazepine. This is associated with altered gene expression and reduced cell proliferation but not severe malformations. Given that even subtle disruptions in early embryonic life can predict morbidity,^127^ it remains important to determine whether the effects observed at young embryonic stages in the mouse model foreshadow an increased incidence of dysmorphologies or morbidity later in life. Indeed, numerous epidemiological studies in humans have shown that EGR, defined as any impairment in fetal growth relative to its expected biological potential in utero, is associated with a greater risk of prenatal morbidity and mortality,^128–130^ as well as predisposing individuals to birth defects and chronic diseases in childhood and adulthood.^131–133^ Noticeably, EGR in humans has been linked to maternal exposure to numerous environmental contaminants, including airborne pollutants, mycotoxins, phthalates, nitrates, and pesticides.^134–141^

Given the evidence from human studies, it is crucial to determine whether the growth restriction that we observed in mice persists throughout gestation. This would help validate the preclinical relevance of our findings to human clinical data.^142^ Finally, the relatively mild growth delay observed in mouse embryos aligns with human exposure scenarios, where there is no reported increase in major birth defects in areas where irrigation relies heavily on treated wastewater containing residual carbamazepine. However, our study suggests that subtle EGR could be occurring, warranting further epidemiological investigation into whether higher incidences of EGR are seen in regions with consistent carbamazepine contamination in water sources.

## Supplementary Material



## Notes

LC/MS/MS analysis on carbamazepine concentrations in female drinking bottles (Figure S1); embryonic stage and somite number measurements in embryos from individual females (Figure S2); flow-cytometry analysis on cell proliferation (Figure S3); morphological criteria in succeeding embryonic stages between E8 and E10 (Table S1); numerical scoring of the heart developmental state in succeeding stages (Table S2); pairwise Spearman correlation matrices between heart scores, embryonic stages and somite numbers (Table S3); R-codes (Supplementary Text 1) (PDF)
