# Supplementary material for: Maternal Exposure to Carbamazepine at Environmentally Relevant Concentrations Causes Growth Delay in Mouse Embryos
Source: ACS Omega. 2025 Aug 14;10(33):37687–701. doi: 10.1021/acsomega.5c04235 (PMC12391942; doi:10.1021/acsomega.5c04235)
Supplement: Supplementary file 1 [file ao5c04235_si_001.pdf]

Title: Maternal exposure to carbamazepine at environmentally-relevant concentrations causes growth delay in mouse embryos.

Authors: Eliane Veretnik [1#], Orit Douek-Maba [1#], Rotem Kalev-Altman [1], Aluma Haiman [1], Maxim Quint [1], Vered Mordehay [2], Neta Shlezinger [1], Yuval Cinnamon [3], Benny Chefetz [2,4], Dalit Sela-Donenfeld [1*.]

Affiliation: (1) Koret School of Veterinary Medicine, The RH Smith Faculty of Agriculture, Food and Environmental Sciences, The Hebrew University of Jerusalem, Rehovot, 76100, Israel. (2) Department of Soil and Water Sciences, The RH Smith Faculty of Agriculture, Food and Environment, The Hebrew University of Jerusalem, Rehovot, 7610001, Israel. (3) Department of Poultry and Aquaculture Science, Institute of Animal Sciences, Agricultural Research Organization - Volcani Institute, Rishon LeZion, 7505101, Israel, (4) Israeli Agriculture Research Organization- Volcani Institute, Rishon LeZion, 7505101, Israel

# Equal contribution

* Corresponding author: dalit.seladon@mail.huji.ac.il

Key words: Tegratol, teratogen, fetus, developmental delay, growth retardation, growth delay, environmental contamination, psychoactive drugs, gene expression, cell proliferation.

Supplementary Figure 1: LC/MS/MS analysis to quantitate carbamazepine concentrations in the drinking bottles of the female mice.

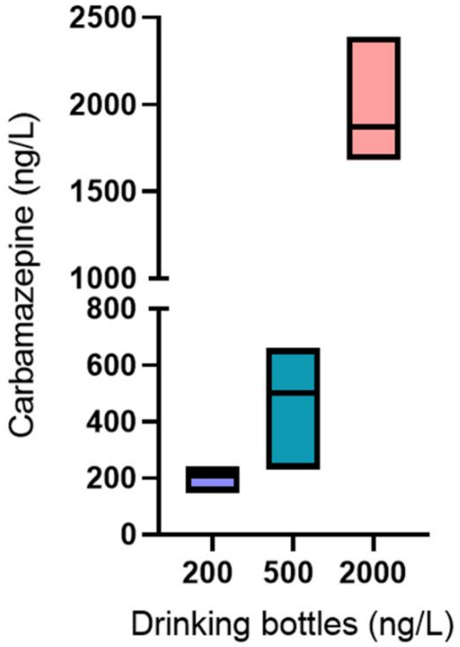

Supplementary Figure 2: Assessment of embryonic stage (A-D) or somite number (E-H) in all embryos of individuals females from the control or carbamazepine groups. Each bar represents a mean value of the measured parameter in all embryos from a single female, named based on her age when harvested and the exposure time to carbamazepine (both in weeks, W).

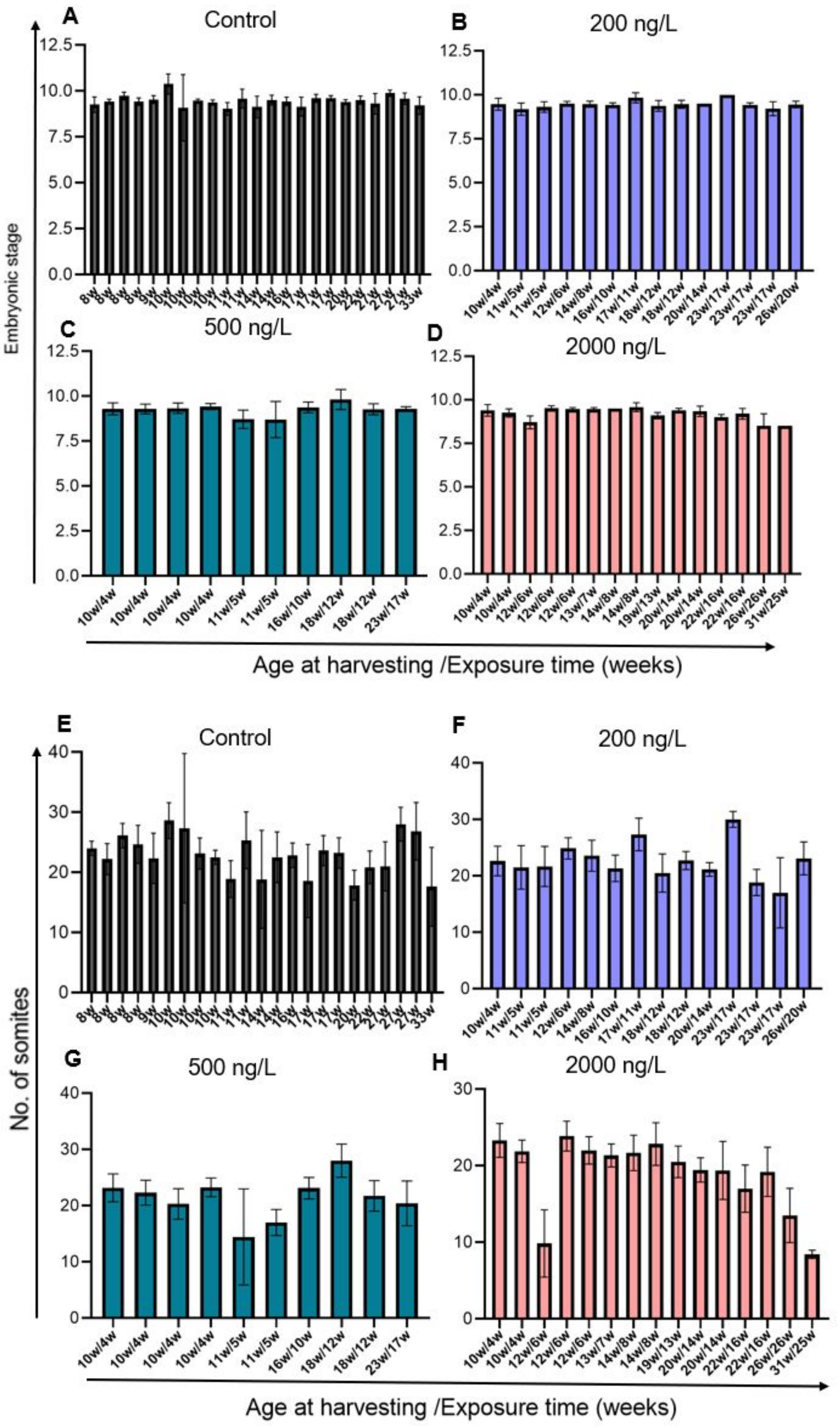

Supplementary Figure 3: Flow-cytometry data from representative experiments to quantify the relative number of fluorescently-labelled PhH3-stained cells out of the total cells in control and carbamazepine-treated embryos. Each raw contained gating of cells from control and carbamazepine- exposed embryos as performed on the same day of analysis. (A, B) Blank samples. (A’, B’) samples from control embryos (A’’, B’’) samples from embryos exposed to 200 ng/Land, 2000 ng/L carbamazepine. FSC-A: forward scatter area.

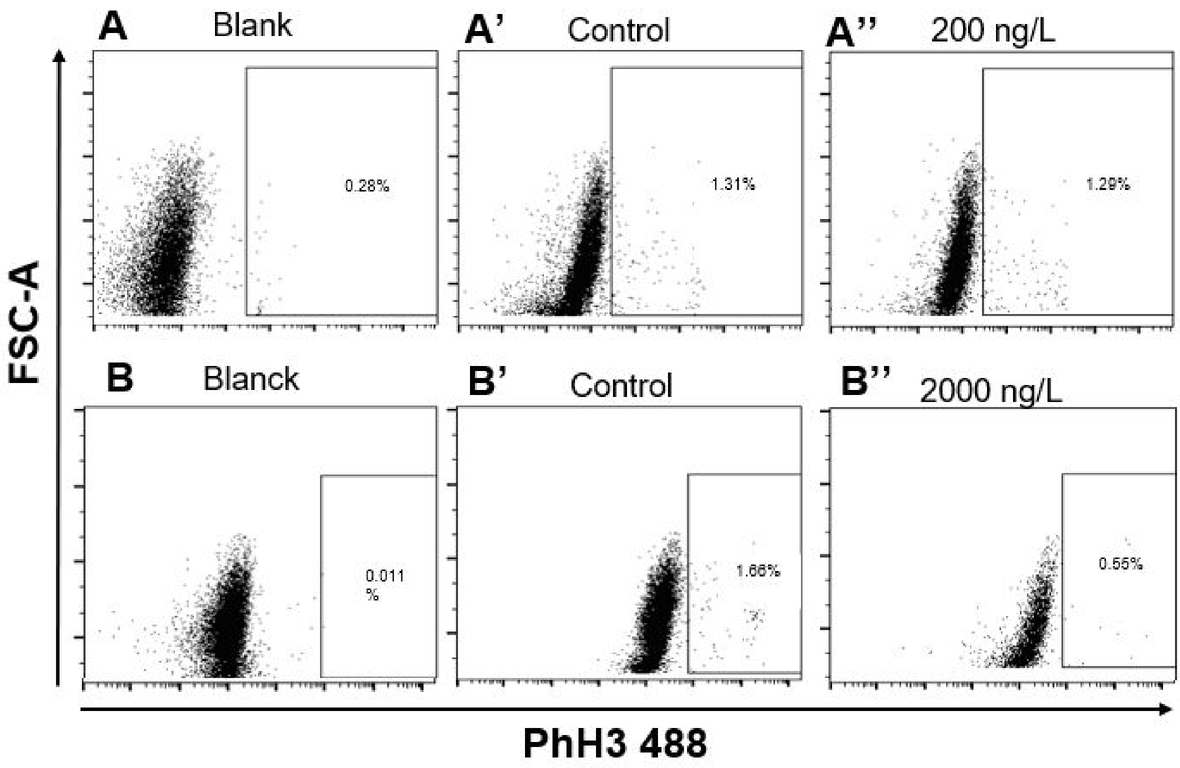

Supplementary Table 1. Morphological criteria corresponding to mouse embryonic developmental progression between E8.0 and E10.0 in quarter-day increments[53,54,68].

Stage	Somite Count	Key Morphological Features
**E8.0**	0–3	Neural plate forms; bilateral cardiac crescent; no turning; no beating; no somites
**E8.25**	4–6	Head fold elevation begins; early heart field migration; no heart tube yet
**E8.5**	7–12	Linear heart tube forms and begins beating; embryo starts turning; 1st pharyngeal arch
**E8.75**	13–16	Head folds deepen; turning nearly complete; 1st and 2nd pharyngeal arches visible
**E9.0**	17–20	Anterior neuropore closes; otic placode visible; 3rd arch forming; C-looping heart
**E9.25**	21–25	Otic pit deepens; tailbud elongates; brain regions expand; early limb bud ridge appears
**E9.5**	26–30	Forelimb bud visible; optic vesicle enlarges; outflow tract lengthens; S-looped heart
**E9.75**	31–34	Hindlimb bud begins; brain vesicles prominent; more defined facial and cardiac structures
**E10.0**	34–40	Full closure of neural tube; paddle-shaped forelimbs; hindlimb bud visible; 4 pharyngeal arches; optic cup and lens vesicle; otic vesicle closed; S-looped heart; elongated tail

Supplementary Table 2. Numerical scoring to quantify the morphlogical state of the heart. Scores 1-4 are based on the sucessive morphologies [70,72,73], as presented from top to bottom:
(1): Bilateral cardiac fields within the cardiac crescent, with no tube formation or beating.
(2) Formation of a primary heart tube in the midline, initiating weak contractions and early rightward looping.
(3) Visible C-looping with bulging of the primitive ventricles and arteries and stronger, peristaltic heartbeats.
(4) Well-defined S-shaped heart with distinct atrial and ventricular regions, an elongated outflow tract, and robust embryonic circulation.

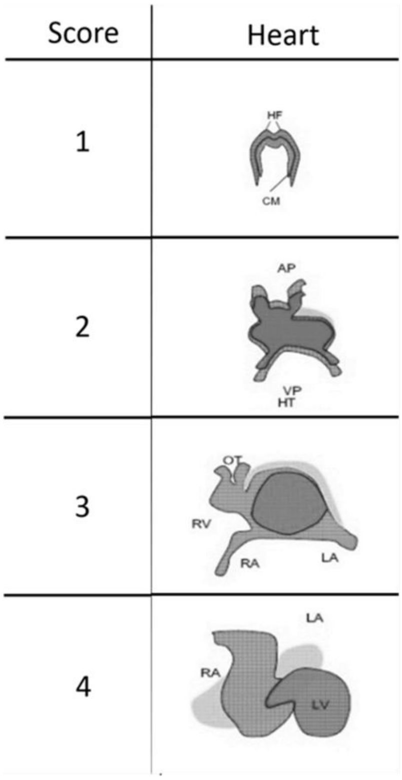

Supplementary Table 3: Pairwise Spearman correlation matrices between heart scores, embryonic stages and somite numbers across all examined groups (control, 200 ng/L, 500 ng/L, and 2000 ng/L carbamazepine), including corresponding p-values for each experimental group,

Control

Parameters	Stage	Somite No.	Heart score
Stage	1	0.77 (p=7.05E-30)	0.63 (p=7.23E-18)
Somite No.	0.77 (p=.7.05E-30)	1	0.44 (p=3.09E-08)
Heart score	0.63 (p=7.23E-18)	0.44 (p=3.09E-08)	1

Carbamazepine 200 ng/L

Parameters	Stage	Somite No.	Heart score
Stage	1	0.71 (p=1.01E-15)	0.49 (p=3.7E-06)
Somite No.	0.71 (p=1.01E-15)	1	0.36 (p=0.000399)
Heart score	0.49 (p=3.7E-06)	0.36 (p=0.000399)	1

Carbamazepine 500 ng/L

Parameters	Stage	Somite No.	Heart score
Stage	1	0.74 (p=4.45E-15)	0.59 (p=7.28E-09)
Somite No.	0.74 (p=4.45E-15)	1	0.55 (p=1.25E-0.6)
Heart score	0.59 (p=7.28E-09)	0.55 p=1.25E-0.6)	1

Carbamazepine 2000 ng/L

Parameters	Stage	Somite No.	Heart score
Stage	1	0.8 (p=2.47E-24)	0.74 (p=6.01E-19)
Somite No.	0.8 (p=2.47E-24)	1	0.74 (p=6.67E-19)
Heart score	0.74 (p=6.01E-19)	0.74 (p=6.67E-19)	1

Supplementary text 1. R code.

````
---

"title: "File_For_Paper
format: html
editor: visual
---

Data Loading and Wrangling ##

{r}```
library(tidyverse)
library(moments)
library(lsr)
library(scales)
install.packages("lsr")#
```

{r}```
somstag = read_csv("somstag2.csv")
somstag = read_csv("somstag.csv")#
```

{r}```
somite = read_csv("somite.csv")#
```

{r}```
stage = read_csv("stage.csv")#
```

Somite ##

Skewness and MAD ###

{r}```
skewness(somstag$somite_control, na.rm = TRUE)
skewness(somstag$somite_200, na.rm = TRUE)
skewness(somstag$somite_500, na.rm = TRUE)
skewness(somstag$somite_2000, na.rm = TRUE)
```

{r}```
aad(somstag$somite_control, na.rm = TRUE)
aad(somstag$somite_200, na.rm = TRUE)
aad(somstag$somite_500, na.rm = TRUE)
aad(somstag$somite_2000, na.rm = TRUE)
```

T - Test ###

{r}```
var.test(somstag$somite_200, somstag$somite_control, alternative = #
"less")
t.test(somstag$somite_200, somstag$somite_control)
print("space")
```
````
````

````

{r}```
var.test(somstag$somite_500, somstag$somite_control, alternative = #
"less")
t.test(somstag$somite_500, somstag$somite_control)
print("space")
```

{r}```
var.test(somstag$somite_2000, somstag$somite_control, alternative = #
"less")
t.test(somstag$somite_2000, somstag$somite_control)
print("space")
```

Somite Discussion ###

{r}```
hist(rbeta(10000,5,2))
hist(rbeta(10000,2,5))
hist(rbeta(10000,5,5))
```

Stage ##

Skewness and MAD ###

{r}```
skewness(somstag$stage_control, na.rm = TRUE)
skewness(somstag$stage_200, na.rm = TRUE)
skewness(somstag$stage_500, na.rm = TRUE)
skewness(somstag$stage_2000, na.rm = TRUE)
```

{r}```
aad(somstag$stage_control, na.rm = TRUE)
aad(somstag$stage_200, na.rm = TRUE)
aad(somstag$stage_500, na.rm = TRUE)
aad(somstag$stage_2000, na.rm = TRUE)
```

T - Test ###

{r}```
var.test(somstag$stage_200, somstag$stage_control, alternative = #
"less")
t.test(somstag$stage_200, somstag$stage_control)
```

{r}```
var.test(somstag$stage_500, somstag$stage_control, alternative = #
"less")
t.test(somstag$stage_500, somstag$stage_control)
```

{r}```
var.test(somstag$stage_2000, somstag$stage_control, alternative = #
"less")
````
````

~~~~
t.test(somstag$stage_2000, somstag$somite_control)
```

Stage Discussion ###

Heart ##

{r}```
var.test(somstag$heart_200, somstag$heart_control, alternative =
"less")
t.test(somstag$heart_200, somstag$heart_control, alternative = "less")
```

{r}```
var.test(somstag$heart_500, somstag$heart_control, alternative =
"less")
t.test(somstag$heart_500, somstag$heart_control, alternative = "less")
```

{r}```
var.test(somstag$heart_2000, somstag$heart_control, alternative =
"less")
t.test(somstag$heart_2000, somstag$heart_control, alternative = "less")
```

Graphs ###

{r}```
library(dplyr)
library(tidyr)
library(ggplot2)

Reshape the data from wide to long format, calculate percentages, and  #
create the bar plot
%<% ggplot(data = somstag
,("pivot_longer(cols = starts_with("heart
,"names_to = "dosage
%<% ("values_to = "heart_stage
mutate(dosage = gsub("heart_", "", dosage)) %>%  # Remove 'heart_'
prefix from dosage
filter(!is.na(heart_stage)) %>%                    # Remove rows with
NA in heart_stage
,mutate(dosage = factor(dosage
,levels = c("control", "200", "500", "2000")
,labels = c("Control", "200", "500", "2000")
ordered = TRUE)) %>%  # Ensure it's treated
as an ordered factor
%<% group_by(dosage, heart_stage)
%<% summarise(frequency = n(), .groups = 'drop')
complete(dosage, heart_stage, fill = list(frequency = 0)) %>% # Fill
in missing combinations with 0
%<% group_by(dosage)
mutate(percentage = (frequency / sum(frequency)) * 100) # Calculate
percentage
+ (
,((geom_bar(aes(x = dosage, y = percentage, fill = factor(heart_stage
+ (()stat = "identity", position = position_dodge
~~~~

```
scale_fill_manual(values = c("black", "gray", "pink", "red")) + # Set
custom colors for heart stages
labs(x = "Dosage Level", y = "Percentage (%)", fill = "Heart Stage")
+
+ ggtitle("Frequency of Heart Stage by Dosage Level")
()theme_minimal
```

```
{r}```
Load necessary libraries #
library(dplyr)
library(tidyr)
library(ggplot2)

Assuming somstag is your data frame and it contains columns for  #
dosage and somite counts
Reshape the data from wide to long format #
%<% long_data <- somstag
pivot_longer(cols = starts_with("somite"),  # Change 'somite' to your
actual column names if needed
,"names_to = "dosage
values_to = "number_of_somites") %>%  # Rename to
reflect somite counts
mutate(dosage = gsub("somite_", "", dosage)) %>%  # Remove 'somite_'
prefix from dosage
filter(!is.na(number_of_somites)) %>%              # Remove rows with
NA in number_of_somites
,mutate(dosage = factor(dosage
,levels = c("control", "200", "500", "2000")
,labels = c("Control", "200", "500", "2000")
ordered = TRUE))  # Ensure it's treated as an
ordered factor

Create stacked histograms for each dosage level #
+ ggplot(data = long_data, aes(x = number_of_somites))
geom_histogram(binwidth = 1, fill = "lightblue", color = "black") + #
Set fill and border colors for clarity
+ labs(x = "Number of Somites", y = "Frequency")
+ ggtitle("Histograms of Somite Counts by Dosage Level")
+ ()theme_minimal
facet_wrap(~ dosage, ncol = 1) # Stack histograms vertically in a
single column
```

```
{r}```
Load necessary libraries #
library(dplyr)
library(tidyr)
library(ggplot2)

Assuming somstag is your data frame and it contains columns for  #
dosage and somite counts
Reshape the data from wide to long format #
%<% long_data <- somstag
pivot_longer(cols = starts_with("somite"),  # Change 'somite' to your
actual column names if needed
,"names_to = "dosage
```

````
values_to = "number_of_somites") %>%  # Rename to
reflect somite counts
mutate(dosage = gsub("somite_", "", dosage)) %>%  # Remove 'somite_'
prefix from dosage
filter(!is.na(number_of_somites)) %>%              # Remove rows with
NA in number_of_somites
,mutate(dosage = factor(dosage
,levels = c("control", "200", "500", "2000")
,labels = c("Control", "200", "500", "2000")
ordered = TRUE))  # Ensure it's treated as an
ordered factor

Create histograms for each dosage level in a single row #
+ ggplot(data = long_data, aes(x = number_of_somites))
geom_histogram(binwidth = 1, fill = "lightblue", color = "black") + #
Set fill and border colors for clarity
+ labs(x = "Number of Somites", y = "Frequency")
+ ggtitle("Histograms of Somite Counts by Dosage Level")
+ ()theme_minimal
facet_wrap(~ dosage, ncol = 4) # Arrange histograms in a single row
with four columns
```

{r}```
library(dplyr)
library(tidyr)
library(ggplot2)

Assuming somstag is your data frame and it contains columns for  #
dosage and somite counts
Reshape the data from wide to long format #
%<% long_data <- somstag
pivot_longer(cols = starts_with("somite"),  # Change 'somite' to your
actual column names if needed
,"names_to = "dosage
values_to = "number_of_somites") %>%  # Rename to
reflect somite counts
mutate(dosage = gsub("somite_", "", dosage)) %>%  # Remove 'somite_'
prefix from dosage
filter(!is.na(number_of_somites)) %>%              # Remove rows with
NA in number_of_somites
,mutate(dosage = factor(dosage
,levels = c("control", "200", "500", "2000")
,labels = c("Control", "200", "500", "2000")
ordered = TRUE))  # Ensure it's treated as an
ordered factor

Create stacked histograms for each dosage level with different colors  #
and transparency
+ ggplot(data = long_data, aes(x = number_of_somites, fill = dosage))
geom_histogram(binwidth = 1, position = "identity", alpha = 0.5,
color = "black") + # Set transparency with alpha
+ labs(x = "Number of Somites", y = "Frequency")
+ ggtitle("Stacked Histograms of Somite Counts by Dosage Level")
+ ()theme_minimal
scale_fill_manual(values = c("lightblue", "lightgreen", "lightcoral",
"lightgoldenrod")) # Custom colors for each dosage level
```
````

```
{r}```
Load necessary libraries #
library(dplyr)
library(tidyr)
library(ggplot2)

Assuming somstag is your data frame and it contains columns for  #
dosage and somite counts
Reshape the data from wide to long format #
%<% long_data <- somstag
pivot_longer(cols = starts_with("somite"),  # Change 'somite' to your
actual column names if needed
,"names_to = "dosage
values_to = "number_of_somites") %>%  # Rename to
reflect somite counts
mutate(dosage = gsub("somite_", "", dosage)) %>%  # Remove 'somite_'
prefix from dosage
filter(!is.na(number_of_somites)) %>%              # Remove rows with
NA in number_of_somites
,mutate(dosage = factor(dosage
,levels = c("control", "200", "500", "2000")
,labels = c("Control", "200", "500", "2000")
ordered = TRUE))  # Ensure it's treated as an
ordered factor

Create smooth density plots for each dosage level with different  #
colors and transparency
+ ggplot(data = long_data, aes(x = number_of_somites, fill = dosage))
geom_density(alpha = 0.5, color = "black", adjust = 1) + # Set
transparency with alpha and smoothness with adjust
+ labs(x = "Number of Somites", y = "Density")
+ ggtitle("Smoothed Density Plots of Somite Counts by Dosage Level")
+ ()theme_minimal
scale_fill_manual(values = c("lightblue", "lightgreen", "lightcoral",
"lightgoldenrod")) # Custom colors for each dosage level
```

{r}```
Load necessary libraries #
library(dplyr)
library(tidyr)
library(ggplot2)

Assuming somstag is your data frame and it contains columns for #
dosage and somite counts
Reshape the data from wide to long format #
%<% long_data <- somstag
pivot_longer(cols = starts_with("somite"), # Change 'somite' to your
actual column names if needed
,"names_to = "dosage
values_to = "number_of_somites") %>% # Rename to
reflect somite counts
mutate(dosage = gsub("somite_", "", dosage)) %>% # Remove 'somite_'
prefix from dosage
filter(!is.na(number_of_somites)) %>%   # Remove rows with
NA in number_of_somites
,mutate(dosage = factor(dosage
```
```

```
,levels = c("control", "200", "500", "2000")
,labels = c("Control", "200", "500", "2000")
ordered = TRUE)) # Ensure it's treated as an
ordered factor

Create smooth density plots for each dosage level in a grid layout #
+ ggplot(data = long_data, aes(x = number_of_somites, fill = dosage))
geom_density(alpha = 0.5, color = "black", adjust = 1) + # Set
transparency with alpha and smoothness with adjust
+ labs(x = "Number of Somites", y = "Density")
+ ggtitle("Smoothed Density Plots of Somite Counts by Dosage Level")
+ ()theme_minimal
facet_wrap(~ dosage, ncol = 1) + # Arrange each density plot in a
single column (stacked)
scale_fill_manual(values = c("lightblue", "lightgreen", "lightcoral",
"lightgoldenrod")) # Custom colors for each dosage level
```

{r}```
} if (!is.numeric(somstag$heart_control))
somstag$heart_control <-
as.numeric(as.character(somstag$heart_control))
{

somstag_control <- somstag[!is.na(somstag$heart_control) &
!is.na(somstag$somite_control) & !is.na(somstag$stage_control),]

Create the plot with direct color assignment in ggplot #
p <- ggplot(data = somstag_control, mapping = aes(x = heart_control, y
+ = somite_control))
geom_jitter(aes(color = factor(stage_control)), width = 0.1, size =
+ 2.0)
)scale_color_manual(values = c
lightpink",   # Very Light Pink" = "8"
FFB3B3",   # Light Pink (smoother transition)#" = "8.25"
FF9999",   # Light Salmon#" = "8.5"
FF7F7F",   # Medium Light Red#" = "8.75"
FFA500",   # Standard Orange#" = "9"
FF8C00",   # Bright Orange#" = "9.25"
FF7F00",   # Deeper Orange#" = "9.5"
FF6600",   # Dark Orange#" = "9.75"
FF5900",   # Very Dark Orange#" = "10"
CC4D00",   # Darker Orange#" = "10.25"
Even Darker Orange #   ,"993300#" = "10.5"
A1A00",   # Dark Red-Orange7#" = "10.75"
C0000"   # Deepest Red (red4)4#" = "11"
+((
+ ggtitle("0mg", "0mg of Drug")
+labs(x = "Heart Level", y = "Somites", color = "Stage")
scale_y_continuous(limits = c(0, 30), breaks = seq(0, 30, by = 5))

ggsave(filename = "somstag_control.png", plot = p, width = 10, height =
6, dpi = 300)
```

{r}```
} if (!is.numeric(somstag$heart_200))
somstag$heart_200 <- as.numeric(as.character(somstag$heart_200))
```

```
{

Filter the data for non-NA values #
somstag_200 <- somstag[!is.na(somstag$heart_200) &
!is.na(somstag$somite_200) & !is.na(somstag$stage_200), ]

)heart_score_1 <- data.frame
,heart_200 = 1
,somite_200 = NA
stage_200 = NA
(

Combine using bind_rows which handles differing column names  #
automatically
somstag_200 <- bind_rows(somstag_200, heart_score_1)

Create the plot with direct color assignment in ggplot #
p <- ggplot(data = somstag_200, mapping = aes(x = heart_200, y =
+ somite_200))
geom_jitter(aes(color = factor(stage_200)), width = 0.1, size =
+ 2.0)
)scale_color_manual(values = c
lightpink",      # Very Light Pink" = "8"
FFB3B3",    # Light Pink (smoother transition)#" = "8.25"
FF9999",     # Light Salmon#" = "8.5"
FF7F7F",    # Medium Light Red#" = "8.75"
FFA500",       # Standard Orange#" = "9"
FF8C00",    # Bright Orange#" = "9.25"
FF7F00",     # Deeper Orange#" = "9.5"
FF6600",    # Dark Orange#" = "9.75"
FF5900",      # Very Dark Orange#" = "10"
CC4D00",   # Darker Orange#" = "10.25"
Even Darker Orange #    ,"993300#" = "10.5"
A1A00",   # Dark Red-Orange7#" = "10.75"
C0000"       # Deepest Red (red4)4#" = "11"
+ ((
+ ggtitle("200mg", subtitle = "200mg of Drug")
+labs(x = "Heart Level", y = "Somites", color = "Stage")
scale_y_continuous(limits = c(0, 30), breaks = seq(0, 30, by = 5))

Save the plot #
ggsave(filename = "somstag_200.png", plot = p, width = 10, height = 6,
dpi = 300)
```

{r}```
} if (!is.numeric(somstag$heart_500))
somstag$heart_500 <- as.numeric(as.character(somstag$heart_500))
{

somstag_500 <- somstag[!is.na(somstag$heart_500) &
!is.na(somstag$somite_500) & !is.na(somstag$stage_500),]

Create the plot with direct color assignment in ggplot #
p <- ggplot(data = somstag_500, mapping = aes(x = heart_500, y =
+ somite_500))
geom_jitter(aes(color = factor(stage_500)), width = 0.1, size =
+ 2.0)
```
```

```
)scale_color_manual(values = c
lightpink",      # Very Light Pink" = "8"
FFB3B3",    # Light Pink (smoother transition)#" = "8.25"
FF9999",     # Light Salmon#" = "8.5"
FF7F7F",    # Medium Light Red#" = "8.75"
FFA500",       # Standard Orange#" = "9"
FF8C00",    # Bright Orange#" = "9.25"
FF7F00",     # Deeper Orange#" = "9.5"
FF6600",    # Dark Orange#" = "9.75"
FF5900",      # Very Dark Orange#" = "10"
CC4D00",   # Darker Orange#" = "10.25"
Even Darker Orange #    ,"993300#" = "10.5"
A1A00",   # Dark Red-Orange7#" = "10.75"
C0000"       # Deepest Red (red4)4#" = "11"
+((
+ ggtitle("500mg", "500mg of Drug")
+labs(x = "Heart Level", y = "Somites", color = "Stage")
scale_y_continuous(limits = c(0, 30), breaks = seq(0, 30, by = 5))

ggsave(filename = "somstag_500.png", plot = p, width = 10, height = 6,
dpi = 300)
```

{r}```
} if (!is.numeric(somstag$heart_2000))
somstag$heart_2000 <- as.numeric(as.character(somstag$heart_2000))
{

somstag_2000 <- somstag[!is.na(somstag$heart_2000) &
!is.na(somstag$somite_2000) & !is.na(somstag$stage_2000),]

Create the plot with direct color assignment in ggplot #
p <- ggplot(data = somstag_2000, mapping = aes(x = heart_2000, y =
+ somite_2000))
geom_jitter(aes(color = factor(stage_2000)), width = 0.1, size =
+ 2.0)
)scale_color_manual(values = c
lightpink",   # Very Light Pink" = "8"
FFB3B3",   # Light Pink (smoother transition)#" = "8.25"
FF9999",   # Light Salmon#" = "8.5"
FF7F7F",   # Medium Light Red#" = "8.75"
FFA500",   # Standard Orange#" = "9"
FF8C00",   # Bright Orange#" = "9.25"
FF7F00",   # Deeper Orange#" = "9.5"
FF7F00",   # Deeper Orange#" = "9.6"
FF6600",   # Dark Orange#" = "9.75"
FF5900",   # Very Dark Orange#" = "10"
CC4D00",   # Darker Orange#" = "10.25"
Even Darker Orange #   ,"993300#" = "10.5"
A1A00",   # Dark Red-Orange7#" = "10.75"
C0000"   # Deepest Red (red4)4#" = "11"
+((
+ ggtitle("2000mg", "2000mg of Drug")
+labs(x = "Heart Level", y = "Somites", color = "Stage")
scale_y_continuous(limits = c(0, 30), breaks = seq(0, 30, by = 5))
```
```

```
ggsave(filename = "somstag_2000.png", plot = p, width = 10, height = 6,
dpi = 300)
```
